# A machine-learning model to identify concurrent vascular disease in symptomatic patients with chronic obstructive pulmonary disease

**DOI:** 10.1080/07853890.2025.2588285

**Published:** 2025-11-21

**Authors:** Yufeng Gu, Ping Chen, Shuhong Wang

**Affiliations:** a Department of Information, Suining Central Hospital, Suining, China; b Department of Neurology, Suining Central Hospital, Suining, China; c Department of Respiratory, Suining Central Hospital, Suining, China

**Keywords:** Machine-learning, prediction model, vascular disease, chronic obstructive pulmonary disease, retrospective study

## Abstract

**Aim/introduction:**

Chronic obstructive pulmonary disease (COPD) is a complex, heterogeneous syndrome often accompanied by vascular diseases that worsen prognosis and quality of life. This study aimed to develop a machine learning model to identify concurrent vascular diseases in symptomatic COPD patients.

**Materials and methods:**

We retrospectively analyzed data from 6,274 COPD patients treated between July 2010 and July 2018. Patients were randomly split into training and validation sets (7:3). After feature selection using LASSO regression, eight machine learning algorithms—including Logistic Regression, Random Forest, Gradient Boosting, Support Vector Machine, Neural Network, Convolutional Neural Network, AdaBoost, and Stacked Generalization (Stacking)—were applied to develop and validate predictive models. Performance was evaluated using AUC, calibration curves, and decision curve analysis (DCA).

**Results:**

The Stacking model achieved the highest AUC (0.867; 95% CI: 0.852–0.882), with 79.4% accuracy, 74.9% sensitivity, and 84.0% specificity. It also demonstrated excellent calibration and, on DCA, provided the highest net clinical benefit within the threshold probability range of 0.1–0.5. At a 0.2 threshold, the model could prevent approximately 35% of unnecessary interventions compared to a "treat-all" approach, while identifying about 75% of high-risk patients relative to a "treat-none" strategy.

**Conclusions:**

The Stacking machine-learning model showed superior performance in identifying concurrent vascular disease among symptomatic COPD patients, offering strong discriminative ability, calibration, and clinical utility. It may serve as an effective decision-support tool to optimize diagnostic evaluation in this high-risk subgroup.

## Introduction

In 2020, approximately 479 million individuals worldwide were affected by chronic obstructive pulmonary disease (COPD), with projections indicating a 23% increase by 2050 [1]. A major contributor to the poor prognosis in COPD is the high prevalence of comorbid vascular diseases, with patients facing a 2.5-fold increased risk of cardiovascular events compared to the general population [2]. These comorbidities, including heart failure (prevalence: 7%–42%), ischemic heart disease (2%–18%), and arrhythmias (3%–21%) account for approximately one-third of COPD-related deaths [3–8]. Notably, this association persists regardless of airflow limitation severity [9], underscoring the critical need to address vascular comorbidities in COPD management [10].

While traditional risk factors like smoking, hypertension, and diabetes are recognized, existing risk prediction models for vascular disease in COPD have relied predominantly on conventional statistical methods [11–13]. These approaches are often limited in their ability to model the complex, non-linear interactions between multiple risk factors and the significant heterogeneity of the COPD population. Consequently, there is a lack of robust, clinically applicable tools for precise individual risk assessment. To address this gap, we leveraged advanced machine learning, which is particularly suited for capturing complex patterns in high-dimensional clinical data.

The novelty of this study lies in the systematic development and comprehensive validation of a suite of machine learning models, including both individual algorithms and a sophisticated Stacking ensemble, specifically for this clinical task. We directly compare the performance of eight distinct algorithms, which, to our knowledge, has not been previously done for identifying vascular disease in COPD. Furthermore, we go beyond mere prediction by employing SHapley Additive exPlanations (SHAP) to ensure model interpretability, thereby identifying and ranking the key clinical drivers of risk. Our primary objective was to create a model with high discriminative power, excellent calibration, and proven clinical utility, as assessed by decision curve analysis, to provide a practical tool for personalizing the management of COPD patients.

## Materials and methods

### Study design and participants

This retrospective diagnostic study included 6,274 adults with clinically diagnosed COPD who presented with symptoms suggestive of vascular disease at our institution between July 2010 and July 2018. Inclusion criteria required documented symptoms of chest tightness, chest pain, or cardiac discomfort. Exclusion criteria comprised incomplete clinical data, severe infections (e.g. sepsis), chronic liver or kidney dysfunction (Child–Pugh ≥ B or estimated glomerular filtration rate <30 mL/min/1.73 m^2^), and immunocompromised status (e.g. HIV/AIDS or immunosuppressive therapy). The hospital ethics committee approved the study, and informed consent was waived due to the retrospective design.

### Data collection and preprocessing

Clinical data were extracted from electronic medical records, including demographics (age and sex), medical history (comorbidities such as hypertension, diabetes, tumors), COPD severity (assessed by GOLD guidelines [14]), and laboratory tests (132 variables encompassing hematological indices [e.g. platelet count, eosinophil ratio], biochemical markers [e.g. LDL/HDL ratio, albumin], coagulation profiles, blood gas analyses, and cardiovascular biomarkers [e.g. myoglobin, cystatin C]). Prior to imputation, we conducted a missingness pattern analysis which indicated no strong evidence of non-random missingness. The majority of variables had less than 5% missing data. Given that the missing data were assumed to be at random (MAR) and the proportion was relatively low, we employed a single imputation approach for simplicity and computational efficiency. We acknowledge that multiple imputation could be an alternative, but for this initial model development study, we opted for median/mode imputation for its simplicity and stability in large datasets. Categorical variables (e.g. hypertension, COPD severity) were label-encoded (0 = no, 1 = yes). Continuous variables were normalized using Z-scores to ensure dimensional consistency. The dataset was randomly stratified into a training set (4,391 cases, 70%) and a validation set (1,883 cases, 30%), maintaining similar positive rates (22.43% vs. 22.41%).

### Outcome definition

Concurrent vascular disease was defined as the presence of: (1) coronary artery disease (CAD), indicated by a history of stable angina, myocardial infarction, percutaneous coronary intervention (PCI), or coronary artery bypass grafting (CABG); and (2) peripheral artery disease (PAD), evidenced by intermittent claudication, relevant surgical history, aortic or leg vessel plaque formation, or aortic intervention. CAD and PAD were combined into a composite “vascular disease” outcome for this analysis. This approach was motivated by the study’s clinical objective: to identify patients with COPD who have a high burden of systemic atherosclerosis, a key prognostic factor. Both CAD and PAD are major clinical manifestations of generalized atherosclerosis and share common pathophysiological pathways relevant to COPD. A composite outcome enhances the clinical utility of the model for overall cardiovascular risk stratification and increases the statistical power for model development. The outcome was assessed based on diagnoses recorded at the time of or prior to the index clinical encounter.

### Feature selection, model training, evaluation and interpretability analysis

Least Absolute Shrinkage and Selection Operator (LASSO) regression with L1 regularization reduced 111 preprocessed features to the most relevant predictors. The regularization parameter λ was optimized *via* 10-fold cross-validation minimizing mean squared error (MSE). Eight algorithms were employed to construct predictive models: Logistic Regression(LR), Random Forest (RF), Gradient Boosting (GB), Support Vector Machine (SVM), Neural Network (NN), Convolutional Neural Network (CNN), AdaBoost, and Stacked Generalization (Stacking). Models were trained on the training set with selected features, and hyperparameters were optimized using grid search and 10-fold cross-validation on the training set. The final set of optimized hyperparameters for each model is provided in Supplementary Table S1.

Receiver operating characteristic (ROC) curves and the area under the curve (AUC) were used to evaluate classification performance. Calibration curves assessed the agreement between predicted probabilities and observed outcomes. Decision Curve Analysis (DCA) evaluated net benefit across probability thresholds, comparing each model with “treat-all” and “treat-none” strategies. SHAP were applied to interpret the optimal Stacking model, with summary plots ranking feature importance by mean absolute SHAP values and force plots illustrating individual prediction contributions. Data preprocessing and model development were conducted in Python (scikit-learn, pandas, Keras, TensorFlow). A detailed list of the software packages and versions is provided in the Supplementary Table S2 to ensure reproducibility.

### Statistical analysis

Patient characteristics were summarized for the training and validation sets. Continuous variables were reported as mean ± standard deviation when normally distributed or median (interquartile range) when non-normally distributed. The Shapiro–Wilk test assessed normality. Between-group differences in continuous variables were analyzed using independent t-tests (normal distribution) or Mann–Whitney U tests (skewed distribution). Categorical variables were presented as frequencies (percentages), with comparisons performed using chi-square tests or Fisher’s exact tests as appropriate. All statistical tests were two-sided, with *p* < 0.05 considered statistically significant. Analyses were conducted using SPSS 26.0 (IBM Corp., Armonk, NY).

## Results

### Baseline characteristics

Among 6,274 patients with COPD, 1,407 patients had vascular disease. The mean age was 69.9 years, and 29.6% were female. Baseline characteristics are presented in Table 1. Most characteristics were balanced between the training and validation sets; however, there was a significant difference in mucous threads (*p* = 0.027).

**Table 1.** Baseline characteristics of included patients.

Variable	Total (*N* = 6274)	Training set (*N* = 4391)	Validation set (*N* = 1883)	*P* value
Age (years)	69.9 ± 9.5	69.9 ± 9.6	70.0 ± 9.5	0.664
Sex, n, %				
Female	1858 (29.6)	1301 (29.6)	557 (29.6)	0.993
Male	4416 (70.4)	3090 (70.4)	1326 (70.4)	
Hypertension, n, %				
Yes	4652 (74.1)	3244 (73.9)	1408 (74.8)	0.477
No	1622 (25.9)	1147 (26.1)	475 (25.2)	
Diabetes, n, %				
Yes	750 (12.0)	524 (11.9)	226 (12.0)	
No	5524 (88.0)	3867 (88.1)	1657 (88.0)	0.973
COPD-level, n, %				
0	554 (8.8)	391 (8.9)	163 (8.7)	0.760
1	2090 (33.3)	1454 (33.1)	636 (33.8)	
2	2600 (41.4)	1812 (41.3)	788 (41.8)	
3	1030 (16.4)	734 (16.7)	296 (15.7)	
Red blood cell	4.3 (3.9–4.7)	4.3 (3.9–4.6)	4.2 (3.9–4.7)	0.824
Neutrophil percentage	73.7 (66.2–80.2)	73.6 (65.7–80.2)	74.0 (67.2–79.9)	0.280
Hematocrit	40.0 (36.8–43.3)	39.9 (36.7–43.2)	40.2 (36.8–43.7)	0.409
Large platelet ratio	37.6 ± 9.9	37.7 ± 9.8	37.3 ± 10.1	0.206
Plateletcrit	0.2 (0.2–0.3)	0.2 (0.2–0.3)	0.2 (0.2–0.3)	0.648
Eosinophil count	0.1 (0.1–0.2)	0.1 (0.0–0.2)	0.1 (0.1–0.2)	0.897
Mean platelet volume	11.6 ± 1.3	11.6 ± 1.3	11.6 ± 1.3	0.229
Platelet count	174.5 (131.0–225.0)	174.9 (131.0–221.7)	174.0 (131.6–233.0)	0.790
Monocyte percentage	7.4 (6.0–9.0)	7.4 (6.0–9.0)	7.5 (5.9–9.1)	0.802
White blood cell count	8.2 (6.3–11.0)	8.2 (6.2–10.9)	8.2 (6.5–11.1)	0.299
Neutrophil count	5.7 (4.1–7.5)	5.7 (4.0–7.5)	5.7 (4.3–7.5)	0.644
Eosinophil percentage	1.5 (0.7–2.9)	1.5 (0.6–2.9)	1.5 (0.7–2.8)	0.758
Monocyte count	0.6 (0.4–0.8)	0.6 (0.4–0.8)	0.6 (0.4–0.8)	0.543
Basophil count	0.0 (0.0-0.0)	0.0 (0.0-0.0)	0.0 (0.0-0.0)	0.969
Platelet distribution width	15.2 ± 3.2	15.2 ± 3.1	15.1 ± 3.3	0.337
Lymphocyte count	1.2 (0.9–1.6)	1.2 (0.9–1.6)	1.2 (0.9–1.6)	0.535
Lymphocyte percentage	17.7 ± 8.0	17.7 ± 8.1	17.6 ± 7.8	0.509
Hemoglobin	128.5 (117.4–139.5)	128.5 (117.0–139.0)	128.8 (118.0–140.5)	0.343
Basophil percentage	0.2 (0.1–0.4)	0.2 (0.1–0.4)	0.2 (0.1–0.4)	0.934
LDL-C/HDL-C	1.6 (1.2–2.1)	1.6 (1.2–2.1)	1.6 (1.2–2.1)	0.411
TG	0.9 (0.7–1.3)	0.9 (0.7–1.3)	0.9 (0.7–1.3)	0.870
Total Protein	67.1 (62.3–72.0)	67.1 (62.3–71.9)	67.1 (62.3–72.3)	0.867
HDL-C	1.4 (1.1–1.7)	1.4 (1.1–1.7)	1.4 (1.1–1.7)	0.655
Total Bilirubin	10.1 (7.5–13.4)	10.1 (7.4–13.5)	10.1 (7.5–13.2)	0.881
Uric Acid	272.5 (211.0–341.0)	270.0 (209.3–340.9)	276.8 (215.9–341.0)	0.300
AST	21.5 (17.0–29.0)	21.0 (17.0–28.5)	21.5 (17.5–29.0)	0.500
Albumin/Globulin ratio	1.3 (1.2–1.5)	1.3 (1.2–1.5)	1.3 (1.2–1.5)	0.804
Serum iron	9.9 (5.8–14.9)	10.0 (6.0–15.1)	9.7 (5.6–14.6)	0.142
Albumin	38.2 (34.7–41.4)	38.2 (34.7–41.3)	38.2 (34.8–41.6)	0.767
Indirect bilirubin	5.8 (4.1–8.1)	5.8 (4.1–8.1)	5.7 (4.0–8.2)	0.622
Gamma-Glutamyl Transferase	27 (18–45)	27 (18–45)	27 (18–46)	0.562
Direct/total bilirubin ratio	0.4 (0.3–0.5)	0.4 (0.3–0.5)	0.4 (0.3–0.5)	0.102
Globulin	29.1 (25.8–32.6)	29.1 (25.8–32.6)	28.9 (25.9–32.4)	0.782
Glucose	5.8 (4.8–7.2)	5.8 (4.9–7.2)	5.7 (4.8–7.3)	0.538
Potassium	3.7 (3.4–4.0)	3.7 (3.4–4.0)	3.7 (3.4–4.0)	0.668
Magnesium	0.8 (0.8–0.9)	0.8 (0.8–0.9)	0.8 (0.8–0.9)	0.970
Creatinine	72 (61–85)	72.0 (60.9–85.0)	72.0 (61.0–86.0)	0.471
Alkaline Phosphatase	70 (59–86)	70.5 (59.0–87.0)	70.0 (58.4–85.0)	0.417
Urea	5.3 (4.2–6.9)	5.3 (4.1–6.8)	5.5 (4.3–7.0)	0.079
Direct bilirubin	3.6 (2.6–5.3)	3.6 (2.6–5.3)	3.7 (2.6–5.4)	0.811
TC	4.3 (3.7–5.0)	4.3 (3.7–5.0)	4.3 (3.6–5.0)	0.166
LDL-C	2.3 (1.8–2.9)	2.3 (1.8–2.9)	2.3 (1.8–2.8)	0.172
ALT	17 (12–27)	17 (12–27)	18.0 (12.9–27.0)	0.611
Erythrocyte sedimentation rate	21 (9–43)	21 (9–43)	21 (9–43)	0.559
Serum cholinesterase	6295.0 (5037.5–7766.5)	6303.2 (5059.5–7783.0)	6284.0 (4976.4–7743.4)	0.381
Apolipoprotein A1	1.4 (1.2–1.6)	1.4 (1.2–1.6)	1.4 (1.1–1.6)	0.332
Lipoprotein(a)	87.0 (30.0–190.0)	84 (30–187)	91.2 (30.9–198.0)	0.138
Carcinoembryonic antigen	2.9 (1.9–4.3)	2.9 (1.9–4.3)	2.9 (1.9–4.2)	0.521
Plasma fibrinogen	4.6 ± 1.6	4.6 ± 1.6	4.7 ± 1.6	0.401
Prothrombin time	13.3 (12.6–14.1)	13.3 (12.6–14.1)	13.3 (12.6–14.1)	0.402
PT activity	98.3 ± 18.7	98.4 ± 18.7	98.1 ± 18.6	0.585
Thrombin time	16.8 (15.9–18.0)	16.8 (15.9–18.0)	16.8 (15.9–18.0)	0.717
Fructosamine	2.0 (1.7–225.0)	2.0 (1.7–226.0)	2.0 (1.7–223.9)	0.173
Myoglobin	43.2 (30.6–65.4)	42.9 (30.6–65.7)	43.8 (30.8–64.9)	0.902
Cystatin C	1.0 (0.9–1.2)	1.0 (0.9–1.2)	1.0 (0.9–1.2)	0.479
Procalcitonin	0.1 (0.1–0.2)	0.1 (0.1–0.2)	0.1 (0.1–0.2)	0.453
pH	7 (6–7)	7 (6–7)	7 (6–7)	0.947
White blood cell count	4.0 (1.9–9.4)	3.9 (1.9–8.9)	4.3 (2.1–10.1)	0.143
Conductivity	16.6 (11.8–21.2)	16.7 (11.7–21.5)	16.4 (11.8–20.6)	0.424
Epithelial cell count	3.5 (1.6–7.0)	3.5 (1.6–7.0)	3.6 (1.6–7.3)	0.649
Crystal examination	0.1 (0.0–0.6)	0.1 (0.0–0.7)	0.1 (0.0–0.6)	0.264
Cast count	0.5 (0.1–1.5)	0.5 (0.1–1.5)	0.6 (0.1–1.6)	0.171
Red blood cell count	6.7 (3.2–14.5)	6.6 (3.2–14.8)	6.7 (3.2–13.7)	0.820
Yeast	0 (0-0)	0 (0-0)	0 (0-0)	0.649
Bacterial count	11.5 (2.8–42.1)	11.2 (2.8–41.2)	12.7 (2.8–44.6)	0.301
Mucous threads	0.7 (0.1–2.7)	0.6 (0.1–2.6)	0.7 (0.1–2.9)	0.027
Pathological casts	0.2 (0.0–0.7)	0.2 (0.0–0.6)	0.3 (0.0–0.7)	0.309
PaO₂	94.0 (74.0–128.0)	93.7 (73.6–128.5)	94.0 (74.5–126.7)	0.667
TCO₂	47.1 ± 17.8	47.3 ± 17.9	46.6 ± 17.8	0.216
HCO₃⁻ concentration	28.8 ± 5.1	28.7 ± 5.1	29.0 ± 5.2	0.095
HCO₃⁻ standard value	26.9 ± 3.3	26.9 ± 3.3	27.0 ± 3.3	0.128
Extracellular Fluid Base Excess	3.8 (1.3–7.3)	3.8 (1.3–7.2)	4.0 (1.4–7.5)	0.202
PaCO₂	48.2 ± 11.0	48.0 ± 10.8	48.6 ± 11.3	0.050
Base Excess	3.1 (1.0–5.8)	3.0 (1.0–5.7)	3.2 (1.1–6.0)	0.245
Immature Granulocyte percentage	0.4 (0.2–0.7)	0.4 (0.3–0.8)	0.4 (0.2–0.7)	0.926
Immature Granulocyte absolute count	0.0 (0.0–0.1)	0.0 (0.0–0.1)	0.0 (0.0–0.1)	0.899
hs-CRP	14.9 (5.9–37.5)	14.4 (6.0–37.0)	15.8 (5.8–37.7)	0.261
D-Dimer	1.2 (0.6–1.9)	1.2 (0.6–1.9)	1.2 (0.6–1.9)	0.824
Monocyte ratio	8.2 (6.7–10.0)	8.2 (6.7–10.0)	8.2 (6.8–9.9)	0.841
RDW-SD	47.4 (44.6–50.5)	47.3 (44.6–50.5)	47.6 (44.6–50.6)	0.311
Neutrophil count	5.3 (3.9–7.0)	5.3 (3.9–7.0)	5.3 (3.9–7.1)	0.895
Neutrophil percentage	71.5 (64.6–77.8)	71.4 (64.6–77.9)	71.7 (64.6–77.5)	0.791
Monocyte count	0.6 (0.5–0.8)	0.6 (0.5–0.8)	0.6 (0.5–0.8)	0.837
RDW-CV	13.8 (13.1–14.6)	13.8 (13.1–14.6)	13.8 (13.1–14.6)	0.848
Cholinesterase	6043.5 (4851.0–7284.8)	6055.5 (4857.5–7323.8)	5973.8 (4834.0–7169.8)	0.129
BUN	5.4 (4.3–7.0)	5.4 (4.3–7.0)	5.5 (4.3–7.0)	0.453
Apolipoprotein B	0.8 (0.7–1.0)	0.8 (0.7–1.0)	0.8 (0.7–1.0)	0.427
INR	1.0 (0.9–1.1)	1.0 (0.9–1.1)	1.0 (1.0–1.1)	0.805
APTT	35.8 (32.2–40.0)	35.9 (32.3–40.0)	35.7 (32.1–40.1)	0.814
α-HBDH	159 (135–193)	160 (136–194)	157.0 (131.0–191.5)	0.066
Troponin I Assay	6.7 (3.0–15.3)	6.7 (3.0–15.1)	6.7 (3.0–15.4)	0.695
NT-proBNP	83.8 (35.2–247.8)	84.3 (34.9–244.0)	83.2 (35.8–256.3)	0.721

### Screening of features

**Figure 1.**
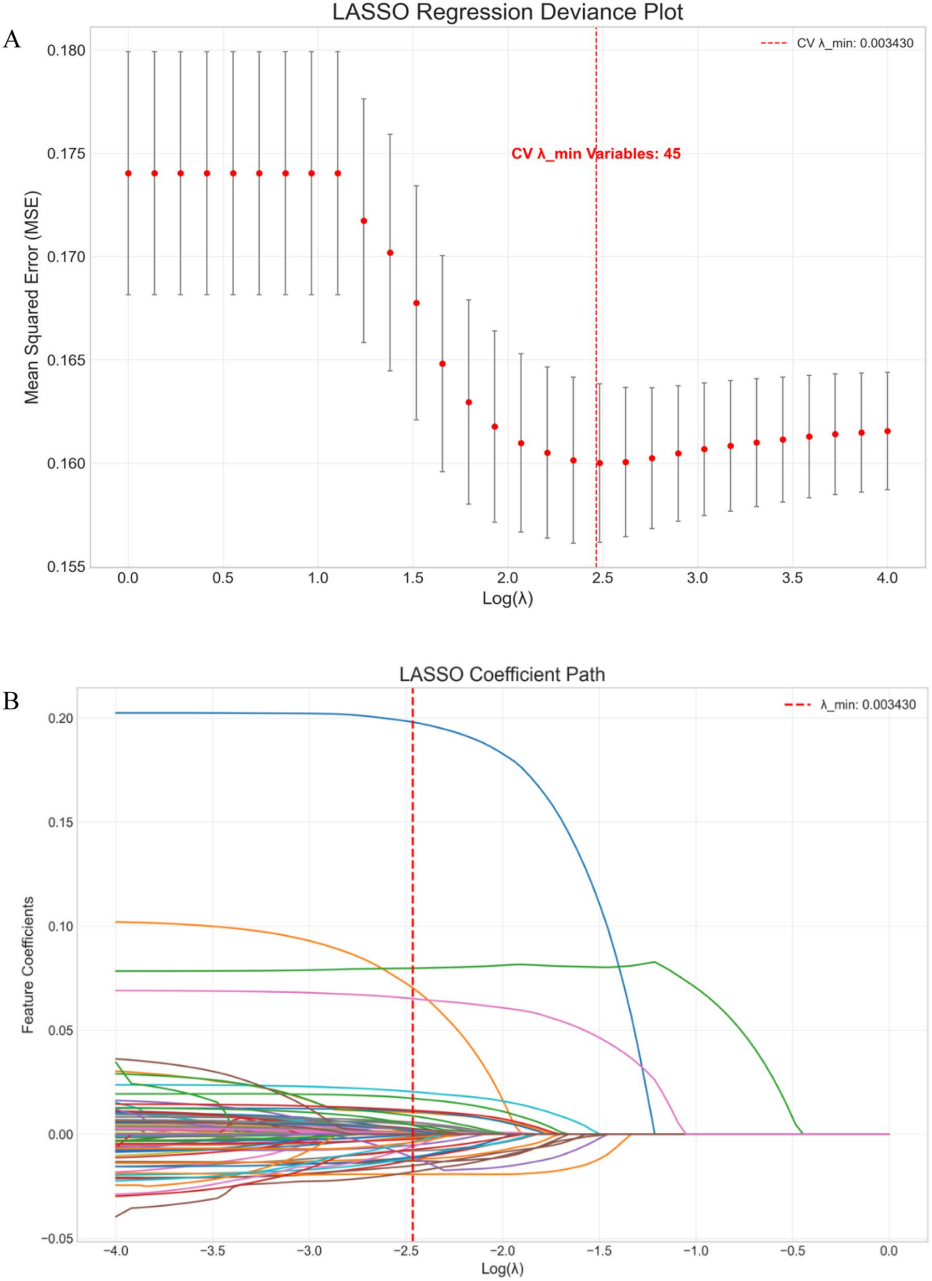
LASSO regression. (A) LASSO regression deviance plot illustrating optimal λ selection. (B) LASSO coefficient path plot depicting feature selection.

All 6,274 samples with 111 feature variables were included in the LASSO regression analysis. The optimal regularization parameter λ was determined using 10-fold cross-validation. In each iteration, nine subsets were used for model training, and the remaining subset evaluated performance. This procedure was repeated 10 times to calculate the mean cross-validation error. The optimal λ value minimized the mean squared error (MSE) and was set at λ = 0.003430. The LASSO deviance plot demonstrated that mean squared error stabilized below this threshold and increased markedly when λ exceeded it, confirming that λ = 0.003430 achieved the best balance between model complexity and predictive accuracy (Figure 1A).

Features were stratified into three tiers based on the absolute values of their LASSO coefficients: high-importance features (|coefficient| >0.01), including 10 features (22.2%); medium-importance features (0.005 < |coefficient| ≤ 0.01), comprising 13 features (28.9%); and low-importance features (0<|coefficient|≤0.005), encompassing 22 features (48.9%). Detailed results appear in Table 2. The LASSO coefficient path plot showed that as λ approached zero, most features retained non-zero coefficients. At λ = 0.003430, 45 features had non-zero coefficients. Increasing λ further progressively shrank these coefficients toward zero. Clinically relevant features, such as hypertension, age, and diabetes, maintained stable non-zero coefficients even at higher λ values (Figure 1B).

**Table 2.** LASSO variable feature importance.

Feature	Coefficient
Hypertension	0.153506617
Age	0.054803061
Diabetes	0.037013592
Sex	0.024279359
Lipoprotein(a)	0.022311072
RDW-SD	0.015337032
TCO₂	0.013926147
Fructosamine	0.012420435
Troponin I	0.012343686
RBC	0.011500288
Plateletcrit	0.009967093
COPD-Level	0.009559369
PaO₂/pO₂	0.009523058
Pathological Casts	0.009372701
Serum Iron	0.009129607
Prothrombin Time	0.00870294
Basophil Percentage	0.008113406
Potassium	0.007770995
APTT	0.00706107
Plasma Fibrinogen	0.006104672
Serum Cholinesterase	0.005300945
LDL-C/HDL-C	0.005263586
HDL-C	0.005232684
Apolipoprotein B	0.004036359
Epithelial Cell Count	0.004027618
Base Excess	0.003515165
Myoglobin	0.002963831
Direct/Total Bilirubin Ratio	0.00295593
Cholinesterase	0.002883878
Crystal Examination	0.002288417
RDW-CV	0.002197852
Hemoglobin	0.002153054
RBC	0.002089006
Lymphocyte Count	0.001875502
Apolipoprotein A1	0.001743526
Neutrophil Count	0.001742719
Albumin/Globulin Ratio	0.001622401
Neutrophil Percentage	0.001406012
Uric Acid	0.001261743
Alkaline Phosphatase	0.001198731
Conductivity	0.001052905
D-Dimer	0.000994124
NT-proBNP	0.000929056
Cystatin C	0.000877729
Magnesium	0.000311562

### Model development and evaluation

The 45 LASSO-selected features were standardized using Z-score normalization to remove scale discrepancies across variables. To align with the distinct characteristics of each algorithm, a second model-specific feature selection was performed using the SelectKBest method based on F-statistics. The optimal number of features k for each algorithm was determined through a tuning process within the training set only. Specifically, for each algorithm, we performed an internal 5-fold cross-validation on the training set, evaluating a range of k values. The k value that yielded the highest mean cross-validation AUC was selected as the final parameter for that algorithm. The final k values chosen for each model were as follows: LR: 20 features; RF: 25 features; GB: 30 features; SVM: 15 features; NN: 20 features; CNN: 30 features; AdaBoost: 20 features; and Stacking: 40 features. This approach ensured that the feature selection was integrated into the model tuning process without peeking at the validation set.

**Figure 2.**
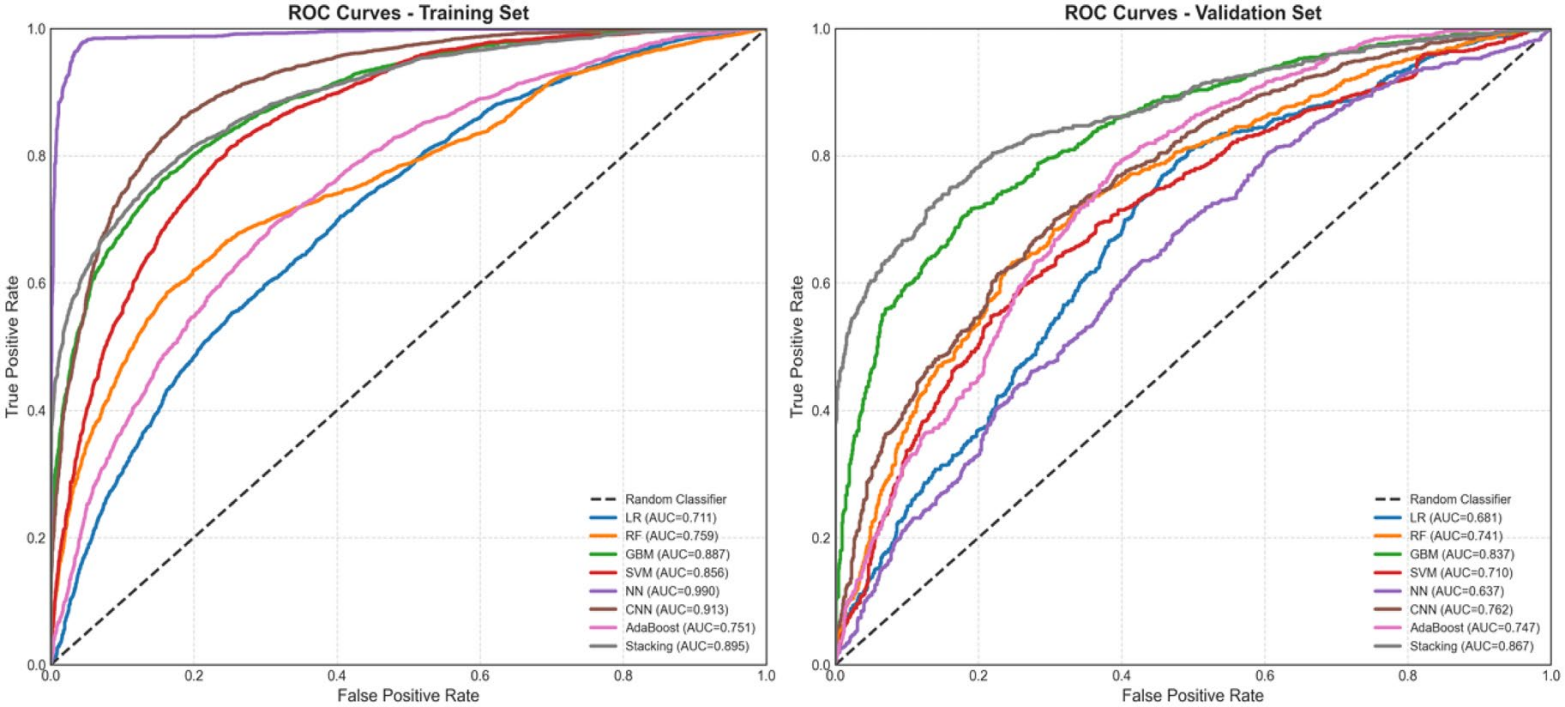
Performance comparison of eight machine learning models in the training and validation sets.

The eight optimized models produced AUC values ranging from 0.683 to 0.867 (mean AUC = 0.761) on the validation set. Two models achieved an AUC >0.80, indicating excellent predictive performance. The Stacking ensemble model yielded the best results, with a validation AUC of 0.867 (95% CI: 0.852–0.882), accuracy of 79.4%, sensitivity of 74.9%, specificity of 84.0%, positive predictive value (PPV) of 82.4%, negative predictive value (NPV) of 77.0%, and F1-score of 78.5%. This model demonstrated strong discriminative ability, accuracy, and robustness (Figure 2 and Table 3).

**Table 3.** The model performance evaluation.

Model	Dataset	AUC	Accuracy	Sensitivity	Specificity	PPV	NPV	F1 Score	Loss
Logistic Regression	Training	0.711	0.635	0.826	0.445	0.598	0.718	0.694	0.651
Random Forest	Training	0.759	0.699	0.694	0.703	0.701	0.697	0.697	0.627
Gradient Boosting	Training	0.887	0.802	0.760	0.845	0.830	0.779	0.794	0.507
SVM	Training	0.856	0.770	0.865	0.675	0.727	0.833	0.790	0.536
**Neural Network**	**Training**	**0.990**	**0.856**	**0.992**	**0.721**	**0.780**	**0.989**	**0.874**	**0.315**
CNN	Training	0.913	0.829	0.898	0.759	0.788	0.882	0.840	0.422
AdaBoost	Training	0.751	0.689	0.683	0.695	0.691	0.687	0.687	0.626
Stacking Ensemble	Training	0.895	0.809	0.768	0.849	0.836	0.786	0.801	0.462
Logistic Regression	Validation	0.708	0.657	0.851	0.463	0.613	0.757	0.713	0.651
Random Forest	Validation	0.734	0.686	0.699	0.672	0.681	0.691	0.690	0.634
Gradient Boosting	Validation	0.837	0.763	0.716	0.810	0.790	0.740	0.751	0.543
SVM	Validation	0.731	0.671	0.710	0.632	0.658	0.685	0.683	0.621
Neural Network	Validation	0.683	0.633	0.695	0.570	0.618	0.652	0.654	0.728
CNN	Validation	0.762	0.696	0.698	0.693	0.695	0.697	0.697	0.581
AdaBoost	Validation	0.765	0.708	0.725	0.691	0.701	0.715	0.713	0.620
Stacking Ensemble	Validation	0.867	0.794	0.749	0.840	0.824	0.770	0.785	0.478

Bold indicates the highest AUC among eight machine learning algorithms.

**Figure 3.**
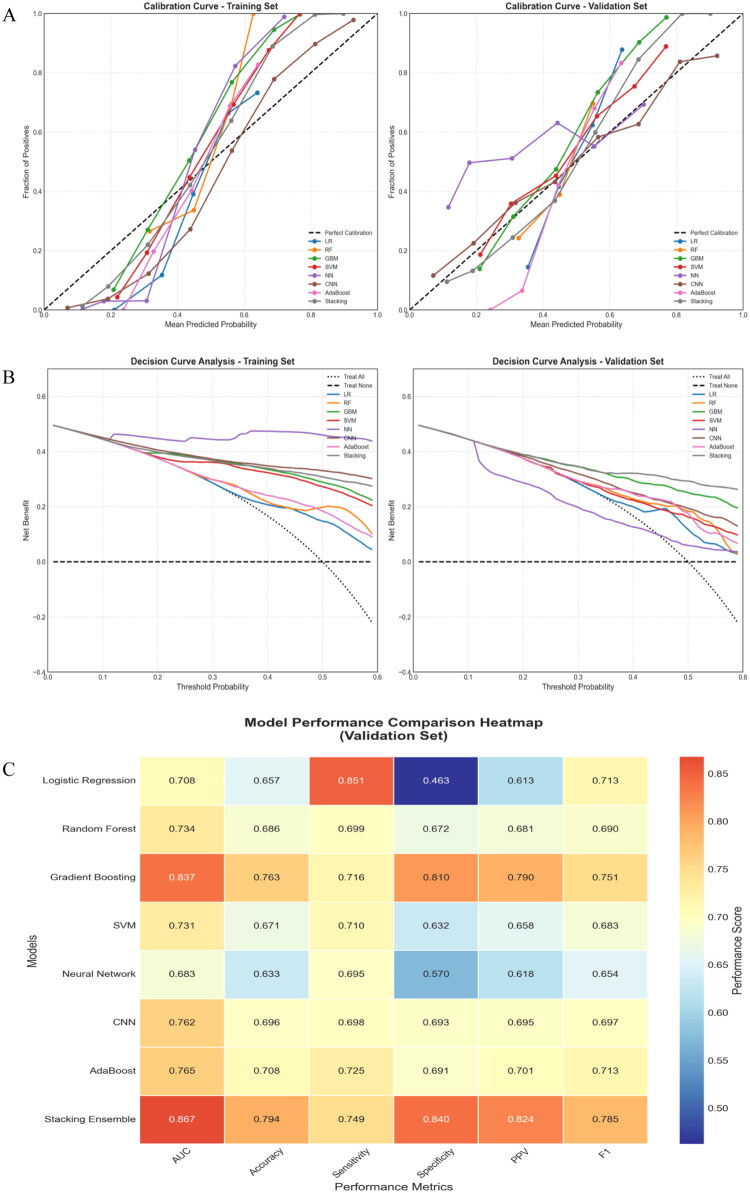
Calibration curve analysis (A), decision curve analysis (B), and model ranking by AUC performance (C).

Calibration curve analysis showed that the Stacking ensemble and Gradient Boosting models provided excellent calibration in the validation set, with predicted probabilities closely following the ideal diagonal line. Although Logistic Regression exhibited good calibration in the training set, it modestly overestimated probabilities in the validation set. The Neural Network model showed miscalibration in low-probability regions, indicating the need for further refinement (Figure 3A). DCA demonstrated that the Stacking model offered the highest clinical net benefit within the 0.1–0.5 threshold probability range. At a 0.2 threshold, this model could reduce approximately 35% of unnecessary interventions compared with a “treat-all” approach while identifying nearly 75% of high-risk patients compared with a “treat-none” strategy. Gradient Boosting also delivered substantial clinical utility within the 0.15–0.4 threshold range (Figure 3B). Models ranked by AUC performance were as follows: Stacking Ensemble (0.867) > GB (0.837) > AdaBoost (0.765) > CNN (0.762) > RF (0.734) > SVM (0.731) > LR (0.708) > NN (0.683). Ensemble methods markedly outperformed individual algorithms (Figure 3C).

**Figure 4.**
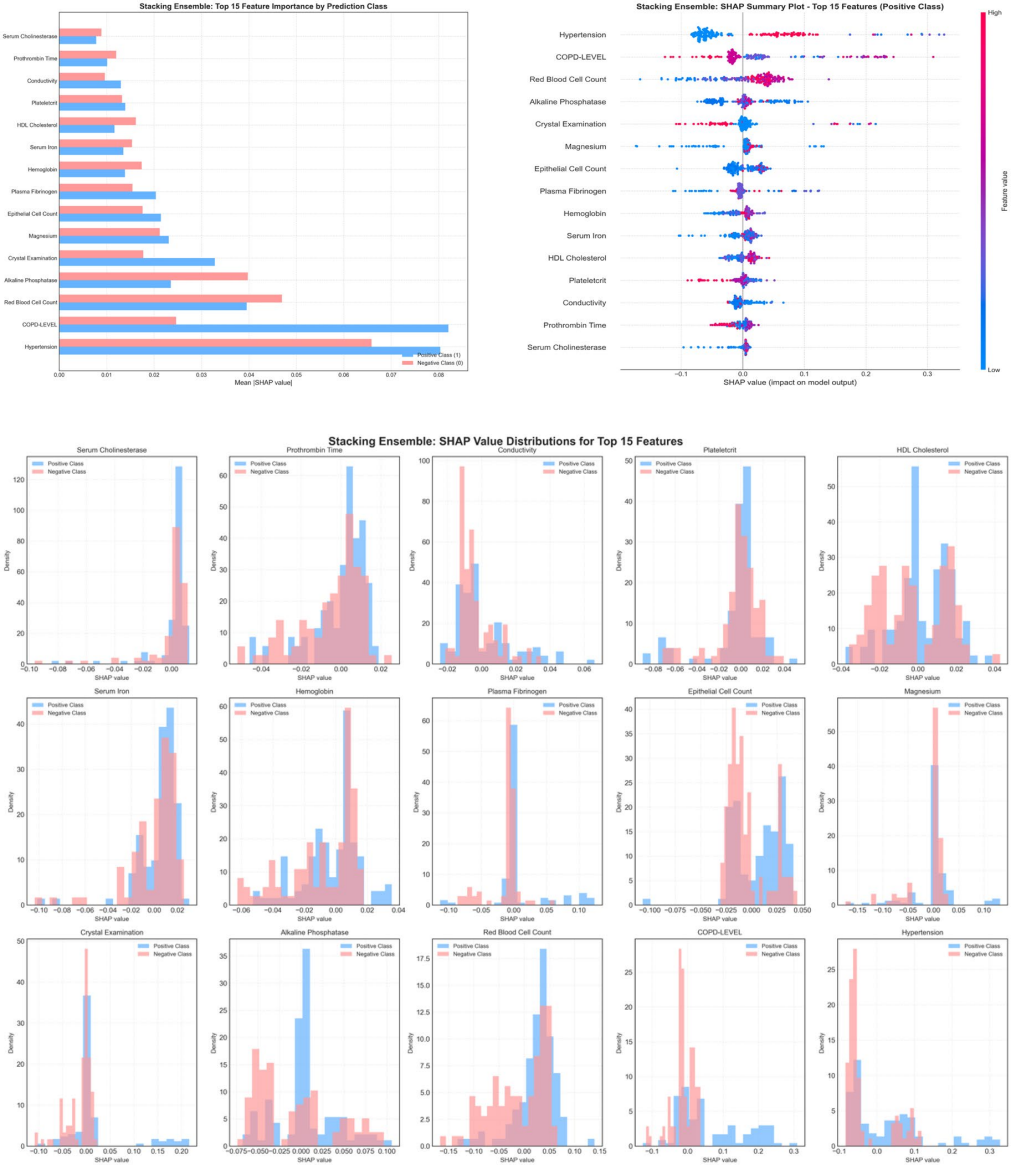
Core predictive features of the Stacking ensemble model (A), SHAP summary scatter plot demonstrating feature impact (B), and class-specific feature importance analysis (C).

The Stacking model identified 15 core predictive features with importance scores ranging from 0.0083 to 0.0737. The five most influential features were hypertension (0.0737), COPD level (0.0556), red blood cell count (0.0430), alkaline phosphatase (ALP) (0.0310), and crystal examination (0.0259) (Figure 4A). The SHAP summary scatter plot illustrated directional effects of features on disease risk. For example, hypertension showed a clear bidirectional pattern: patients with hypertension (red dots) clustered in regions with positive SHAP values (higher risk), while patients without hypertension (blue dots) clustered in regions with negative SHAP values (lower risk) (Figure 4B).

Class-specific feature importance analysis indicated that hypertension had much higher importance in the positive class (disease group, 0.065) compared to the negative class (control group, 0.010), confirming its role as a disease risk factor. COPD level similarly contributed more prominently in the disease group. Hematological indicators, such as red blood cell count, maintained relatively balanced importance across both classes, suggesting their value as continuous predictors of risk (Figure 4C).

## Discussion

This study developed and validated a machine learning model to identify concurrent vascular disease in a cohort of symptomatic patients with COPD. The findings demonstrate that the Stacking ensemble model achieved strong performance in distinguishing patients with COPD and comorbid vascular disease (validation set AUC = 0.867), significantly outperforming seven other machine learning algorithms (GB, AdaBoost, CNN, RF, SVM, LR, NN). The model also showed superior calibration and clinical utility. These results align with previous studies indicating that ensemble methods enhance predictive accuracy by integrating diverse model strengths [15,16]. Calibration curves and DCA confirmed that the Stacking model possesses robust probability calibration and clinical net benefit, supporting reliable stratification of patients into risk categories to guide clinical decisions. This model offers a data-driven tool to improve management of COPD—a complex, heterogeneous syndrome—by targeting its prevalent vascular complications.

Our findings are consistent with a growing body of literature demonstrating the superior performance of machine learning models over traditional methods in predicting vascular diseases. For instance, Yildirim et al. showed that an XGBoost model based solely on ECG waveform features from treadmill tests could predict obstructive coronary artery disease (AUC = 0.78) more accurately than cardiologists’ interpretation, highlighting machine-learning’s ability to extract predictive signals from complex physiological data without relying on conventional clinical markers [17]. Similarly, a multimodal deep learning model for predicting short-term mortality in acute pulmonary embolism achieved remarkable performance (AUC = 0.98) by integrating imaging data with clinical variables, significantly outperforming the standard PESI score [18]. These studies, along with our own, underscore a paradigm shift towards data-driven diagnostics. However, our work distinctively focuses on the long-term, comorbidity-based risk stratification of vascular disease within a specific, high-risk population—patients with COPD. Unlike models designed for acute events or specific diagnostic procedures, our model leverages a wide array of routinely collected clinical and laboratory data to forecast the development of broader vascular comorbidities, emphasizing prevention and personalized management in chronic disease care.

The Stacking ensemble model, which combined GB, RF, LR, and SVM base learners, effectively captured nonlinear interactions among predictors of COPD and vascular disease [19,20]. Its validation set AUC (0.867) exceeded typical performance of traditional risk scores in comparable populations. The model demonstrated accurate calibration, with predicted probabilities closely aligning with observed outcomes across the risk spectrum. This precision is essential for clinical risk assessment. DCA showed that the model provided the highest net benefit within threshold probabilities of 0.1–0.5. At a 20% threshold, it could prevent approximately 35% of unnecessary interventions compared to a “treat all patients” approach while identifying about 75% of high-risk patients compared to a “treat none” strategy. This enables more efficient allocation of healthcare resources and precise targeting of preventive measures.

To better contextualize the performance of our Stacking model (AUC = 0.867), it is important to consider it relative to existing clinical risk scores. Widely used general cardiovascular risk scores, such as the Framingham Risk Score or ASCVD Risk Estimator [21,22]. While a direct head-to-head comparison within our cohort was not feasible due to the lack of required variables, our model’s superior AUC suggests a potential advantage in the specific subpopulation of patients with COPD. This advantage likely stems from our model’s incorporation of COPD-specific factors and a broader set of routinely available clinical biomarkers that capture the systemic manifestations of COPD, which are not fully accounted for in traditional scores. Therefore, the primary novelty and utility of our model lie not necessarily in outperforming these scores in a general sense, but in providing a tailored risk assessment tool that integrates respiratory and systemic health data specifically for the high-risk COPD population.

The SHAP analysis provided critical insights into the feature importance, with some predictors like hypertension [23,24] and COPD severity [25–27] having well-established links to vascular disease. However, the prominence of other biomarkers, such as ALP and urinary crystal examination, warrants further discussion as they may reflect underappreciated pathophysiological pathways. Elevated ALP, often regarded as a marker of liver or bone turnover, has emerged in recent literature as a surrogate marker of chronic systemic inflammation and vascular calcification [28,29]. In the context of COPD, persistent systemic inflammation can stimulate ALP activity in vascular smooth muscle cells, promoting arterial stiffness and atherosclerosis [30]. Thus, ALP may serve as an integrative biomarker capturing the burden of extra-pulmonary systemic effects of COPD that contribute to vascular injury. Similarly, the presence of crystals in urine, while non-specific, can be an indicator of metabolic dysregulation, such as acidosis or electrolyte imbalances [31]. COPD patients frequently have chronic respiratory acidosis and altered renal acid-base handling, which can predispose them to crystal formation [32]. Furthermore, metabolic syndrome—a known risk factor for vascular disease—is also associated with specific crystalluria [33]. Therefore, this variable might act as a proxy for underlying metabolic disturbances that are shared risk factors for both COPD progression and vascular endothelial dysfunction. The model’s selection of these features underscores the complex, multi-systemic nature of the COPD-vascular disease relationship, moving beyond traditional cardiopulmonary risk factors.

It is crucial to interpret the performance and application of our model within the context of its design. Our study population was enriched for the outcome of interest, as we specifically included COPD patients who presented with symptoms suggestive of vascular disease. Therefore, the model’s high performance (AUC = 0.867) reflects its ability to differentiate between those with and without concurrent vascular disease within this high-risk symptomatic subgroup. It does not represent the performance that could be expected if the model were applied to an asymptomatic, general COPD population for predicting future incident disease. This design choice, while limiting the generalizability of our findings for screening purposes, enhances the model’s potential clinical utility as a triage tool in a relevant clinical scenario: helping clinicians decide which symptomatic COPD patients warrant more intensive or urgent cardiovascular diagnostic testing. Future research should aim to develop models for predicting incident disease in broader COPD cohorts, but our study addresses the immediate need of managing complex, symptomatic patients.

The findings also highlight limitations of traditional statistical methods in characterizing complex, multi-factorial associations. Single algorithms such as LR and NN demonstrated inferior performance, likely due to challenges in modeling intricate feature interactions. In contrast, ensemble methods like Stacking and GB effectively integrated multi-dimensional data, emphasizing the value of machine learning in precision health. However, this study has several limitations. First and most fundamentally, the study design introduces a critical limitation: the inclusion of only patients with symptoms suggestive of vascular disease. Because these symptoms are intrinsically linked to the outcome, the model is not predicting incident vascular disease in a general COPD population. Instead, its performance demonstrates an ability to identify concurrent vascular disease within a group already enriched for the condition. This inevitably inflates the apparent performance metrics compared to a true screening scenario and defines the specific clinical context for which our model is relevant. Second, its retrospective, single-center design may introduce selection bias and limit generalizability. Third, reliance on historical electronic medical record data, despite imputation, could compromise data accuracy and completeness. Future research should validate the model in multi-center prospective cohorts and improve its predictive power by incorporating longitudinal biomarker data. Fourth, deploying the model in clinical practice requires addressing challenges, including explaining model outputs to clinicians, integrating the model into workflows, and mitigating potential fairness concerns. Fifth, while internal validation showed promising results, our model requires external validation in multi-center, prospective cohorts with different demographic and clinical characteristics to truly assess its generalizability and robustness before clinical implementation. Sixth, despite employing rigorous measures to prevent overfitting—including a 70/30 split for training/validation, feature selection using LASSO regression strictly on the training set, and 10-fold cross-validation for hyperparameter tuning—we acknowledge that the high performance of the complex Stacking ensemble could still be susceptible to some degree of overfitting to the patterns within our single-center dataset. This risk underscores the critical necessity of external validation in multi-center, prospective cohorts to confirm the model’s generalizability and true predictive power before any clinical application can be considered. Seventh, our composite outcome of “vascular disease,” which combined CAD and PAD, while clinically meaningful for assessing generalized atherosclerotic risk, is a limitation. CAD and PAD have distinct pathophysiological elements, and combining them may obscure specific predictor-outcome relationships. A model trained specifically on CAD or PAD might reveal different and important feature sets. Unfortunately, due to sample size limitations and significant comorbidity between CAD and PAD in our retrospective cohort, a robust separate analysis for each condition was not feasible. Eighth, although the dataset was randomly split into training and validation sets, one variable (‘Mucous threads’) showed a statistically significant difference between the two sets. This highlights the challenge of achieving perfect balance on all covariates in a single randomization. However, given that this variable was not among the top predictors selected by the LASSO regression and subsequent feature selection steps, its influence on the final model’s predictive performance is likely negligible. Future studies could consider more advanced splitting techniques, such as propensity score matching, to ensure better covariate balance if a specific variable is deemed critically important. Ninth, our use of median/mode imputation for handling missing data is a limitation. Although the proportion of missingness was low for each variable, this method is simplistic and can lead to biased estimates and underestimated variance by not accounting for the uncertainty of the missing values. More sophisticated approaches, such as Multiple Imputation by Chained Equations, would provide a more robust handling of missing data. The choice of median/mode imputation was based on computational practicality for this initial model development study. Finally, an analysis on an asymptomatic cohort would be invaluable for contextualizing the model’s performance. However, such an analysis was not feasible in this study due to the lack of a sufficiently sized asymptomatic patient group in our database, which was structured around symptomatic presentation.

Looking forward, our study contributes to the expanding landscape of artificial intelligence in cardiovascular medicine, a field poised to redefine chronic disease management [34]. As emphasized in a recent review, in an increasingly digitalized health industry, the critical step is not to resist artificial intelligence but to adopt it to gain a deeper, patient-level understanding of complex conditions [34]. Our model exemplifies this potential for coronary artery disease and other vascular comorbidities in the high-risk COPD population. For such artificial intelligence systems to realize their full impact, the focus must shift beyond mere model development to seamless integration into clinical workflows. This entails building physician trust through interpretable models, ensuring robust data governance, and addressing potential biases. Furthermore, as artificial intelligence becomes more prevalent, preparing clinicians for this shift is paramount. Physicians will need to acquire the skills to critically appraise artificial intelligence tool outputs and appropriately integrate data-driven insights with their clinical expertise to make informed, personalized patient management decisions [34].

## Conclusion

In summary, this study developed and validated a Stacking ensemble machine learning model with high discriminative ability, excellent calibration, and substantial clinical utility for identifying concurrent vascular diseases in symptomatic COPD patients. Using routinely available clinical data, the model identified key predictors—including hypertension, COPD severity, and specific biomarkers—and provided insights into the mechanisms underlying COPD–vascular disease coexistence. If prospectively validated, the integration of such models into clinical workflows represents a step towards the future envisioned for artificial intelligence in chronic disease management. This requires not only technological validation but also a paradigm shift in clinical practice, where physicians are equipped to leverage artificial intelligence-derived insights for personalized vascular risk assessment, thereby guiding targeted preventive strategies and ultimately improving outcomes in this high-risk population.

## Supplementary Material

Table S1.docx

Table S2.docx

## Data Availability

The datasets used and/or analyzed during the current study are available from the corresponding author upon reasonable request
