# Supplementary material for: A machine-learning model to identify concurrent vascular disease in symptomatic patients with chronic obstructive pulmonary disease
Source: Ann Med. 2025 Nov 21;57(1):2588285. doi: 10.1080/07853890.2025.2588285 (PMC12642907; doi:10.1080/07853890.2025.2588285)
Supplement: Table S1.docx [file IANN_A_2588285_SM5595.docx]

Supplementary Table S1: Optimized Hyperparameters for Machine Learning Models

| **Model** | **Key Hyperparameters** | **Optimized Value** |
| --- | --- | --- |
| Logistic Regression (LR) | Penalty | L2 (Ridge) |
|  | Regularization parameter (C) | 1.0 |
|  | Solver | liblinear |
| Random Forest (RF) | Number of estimators (n_estimators) | 200 |
|  | Maximum depth (max_depth) | 15 |
|  | Minimum samples split (min_samples_split) | 5 |
| Gradient Boosting (GB) | Number of estimators (n_estimators) | 300 |
|  | Learning rate | 0.1 |
|  | Maximum depth (max_depth) | 4 |
| Support Vector Machine (SVM) | Kernel | Radial Basis Function (RBF) |
|  | Regularization parameter (C) | 10 |
|  | Kernel coefficient (gamma) | scale |
| Neural Network (NN) | Hidden layer sizes | (100, 50) |
|  | Activation function | ReLU |
|  | Solver | Adam |
|  | L2 regularization (alpha) | 0.001 |
| Convolutional Neural Network (CNN) | Architecture | Based on reference [X] * |
|  | Number of convolutional layers | 2 |
|  | Optimizer | Adam |
|  | Learning rate | 0.001 |
| AdaBoost | Base estimator | Decision Tree (max_depth=1) |
|  | Number of estimators (n_estimators) | 100 |
|  | Learning rate | 0.8 |
| Stacking Ensemble | Base learners | GB, RF, LR, SVM |
|  | Meta-learner | Logistic Regression (C=1.0) |
|  | Cross-validation for meta-features | 5-fold |
