# Supplementary material for: A machine-learning model to identify concurrent vascular disease in symptomatic patients with chronic obstructive pulmonary disease
Source: Ann Med. 2025 Nov 21;57(1):2588285. doi: 10.1080/07853890.2025.2588285 (PMC12642907; doi:10.1080/07853890.2025.2588285)
Supplement: Table S2.docx [file IANN_A_2588285_SM5594.docx]

Supplementary Table S2: Software and version

| **Package** | **Version** | **Purpose** |
| --- | --- | --- |
| Python | 3.9.12 | Programming language |
| pandas | 1.5.3 | Data manipulation and analysis |
| numpy | 1.23.5 | Numerical computations |
| scikit-learn | 1.2.2 | Machine learning (LASSO, models, metrics) |
| TensorFlow/Keras | 2.11.0 | Neural network models (NN, CNN) |
| SHAP | 0.41.0 | Model interpretability |
| matplotlib | 3.7.0 | Plotting and visualization |
| seaborn | 0.12.2 | Statistical data visualization |
